# Elucidation of the Role of SMAD4 in Epithelial–Mesenchymal Plasticity: Does It Help to Better Understand the Consequences of *DPC4* Inactivation in the Malignant Progression of Pancreatic Ductal Adenocarcinoma?

**DOI:** 10.3390/cancers15030581

**Published:** 2023-01-18

**Authors:** Hendrik Ungefroren, Björn Konukiewitz, Rüdiger Braun, Ulrich Friedrich Wellner, Tobias Keck, Jens-Uwe Marquardt

**Affiliations:** 1 First Department of Medicine, University Hospital Schleswig-Holstein (UKSH), Campus Lübeck, D-23538 Lübeck, Germany; 2 Institute of Pathology, University Hospital Schleswig-Holstein, Campus Kiel, D-24105 Kiel, Germany; 3 Center of Brain, Behavior and Metabolism (CBBM), University of Lübeck, D-23538 Lübeck, Germany; 4 Department of Surgery, University Hospital Schleswig-Holstein, Campus Lübeck, D-23538 Lübeck, Germany

Pancreatic ductal adenocarcinoma (PDAC) is the 4th leading cause of cancer-related mortality worldwide, with a 5-year-survival rate below 10% that is the lowest of all cancer types. Its incidence is predicted to become the second leading cause of cancer deaths in 2030. The extremely poor prognosis is due to late diagnosis, the lack of specific symptoms and of diagnostic or prognostic tumor markers. As a result of early metastatic spread, only 10–20% are amenable to surgery at the time of diagnosis. The most commonly employed therapies for patients with unresectable or borderline resectable PDAC include (neoadjuvant) chemotherapy and/or radiotherapy, which, however, provide only little survival benefit due to the intrinsic resistance of pancreatic tumors to chemo-, radio- or targeted molecular therapy. More effective therapies, particularly targeted therapies, and the identification of subgroups of patients who could benefit from individualized therapies are, therefore, desperately needed. This, in turn, requires a better understanding of the molecular mechanisms that promote metastatic dissemination and chemoresistance. The four main driver genes of PDAC are oncogenic *KRAS* (mutated in almost 95% of PDACs) along with alterations in the tumor-suppressor genes *CDKN2A*, *TP53* and *DPC4*. These mutations are acquired successively over decades, with *KRAS* and *CDKN2A* being affected early in the carcinogenesis, and *TP53* and *DPC4* later, predominantly in invasive PDAC. *DPC4* is inactivated in 50–55% of patients by different mutational events, mainly homozygous deletion, mutations leading to a non-functional protein together with loss of the wild-type allele, and loss of heterozygosity of 18q (where *DPC4* is located).

*DPC4* encodes SMAD4, a member of the Smad family of transcription factors and central signal transducer of the transforming growth factor (TGF)-β signaling pathway. Following TGF-β binding to its receptors, SMAD4 translocates with SMAD2 or 3 to the nucleus to regulate transcription of responsive target genes. This mode of signaling is referred to as the canonical TGF-β pathway, while in the absence of SMAD2/3/4, TGF-β can also activate non-canonical signaling in a Smad-independent fashion through the mitogen-activated protein kinases, ERK, JNK or p38, and/or the small GTPases RAS or RAC1. In epithelial cells, the TGF-β pathway has a dual role, functioning both as a tumor suppressor in normal cells and at early stages of tumor development and a tumor promoter at later stages. It has been suggested that the canonical TGF-β pathways plays a tumor-suppressive role mainly based on its potent cytostatic effects, while non-canonical signaling promotes malignant behavior. TGF-β can also restrict tumor formation by acting on the surrounding stroma. However, when the TGF-β switch takes place, the composition of the tumor microenvironment is modulated in a way that promotes tumor cell proliferation, migration, invasion and chemoresistance. For instance, in a genetically engineered mouse model (GEMM), TGF-β through the TP53 homolog, TP73, and SMAD4 stimulates expression and secretion of the small proteoglycan biglycan. This pericellularly located protein binds to TGF-β, blocks its interaction with signaling receptors, and along with persistent SMAD4 expression prevents a shift from tumor-suppressive canonical to prometastatic non-canonical TGF-β signaling [1].

It is important to point out that SMAD4 signaling can be triggered by inputs other than TGF-β, i.e., activin, nodal, or myostatin. Further, SMAD4 is also the central mediator of the bone morphogenetic protein (BMP) branch. While the TGF-β pathway is often disrupted in PDAC, alterations in the BMP pathway have not been frequently reported and its role in PDAC remains poorly understood. BMP proteins are capable of inducing epithelial–mesenchymal transition (EMT) in pancreatic cell lines via functional SMAD4 and in human colorectal cancer models of EMT, BMP signaling for EMT execution via SNAIL requires SMAD4 in a TGF-β-independent manner [2]. However, inactivation of BMP receptors has also been associated with increased invasiveness and poor prognosis, which would be consistent with the ability of BMP7 to antagonize EMT and/or induce the reverse process, mesenchymal-epithelial transition (MET), in melanoma cells [3].

TGF-β is one of the strongest inducers of EMT, a process through which epithelial cells acquire an invasive or metastatic phenotype. EMT has long been viewed as a binary process with only one extreme epithelial and mesenchymal phenotype, but recent research suggests that the EMT process comprises a spectrum of intermediary states through which the cells transit in a reversible manner, with each state co-expressing a specific mixture of epithelial and mesenchymal markers and having its own characteristics and behavior [4]. Most notably, cells passing through these intermediate states are characterized by a higher tumor-initiation potential and metastatic abilities than those on the more extreme ends of the EMT spectrum. This refined type of EMT, previously called hybrid, partial or intermediate EMT, is now increasingly referred to as epithelial-mesenchymal plasticity (EMP) [5]. Intriguingly, even a lethal form of EMT has been described that is independent of TGF-β but nevertheless induced by SMAD4 and considered to be another tumor-suppressive function of *DPC4* [6].

It has previously been observed in patients with PDAC that both SMAD4 loss and EMT/EMP were independently associated with enhanced invasion and metastasis; however, whether loss of SMAD4, or other alterations leading to a non-functional SMAD4 protein, have a more direct role in the regulation of EMP is currently not clear. Racu and coworkers summarized and analyzed the current knowledge on this issue and speculated that *DPC4* could represent the functional link between the metastatic process and EMP [7]. Before the authors discuss this in detail, they briefly review the influence of SMAD4 alterations on survival and therapy resistance, stating that several large retrospective studies were unable to demonstrate a prognostic impact on either OS or DFS. In other studies, *DPC4* inactivation was a negative prognostic factor solely in a specific subset of patients, i.e., only homozygous deletions but not *DPC4* mutations were associated with worse DFS. The authors concluded that further investigations are needed to understand the prognostic value of *DPC4* alterations in PDAC.

Since EMT has been associated with cellular mechanisms of invasion and metastasis, data on the role of *DPC4* alterations in PDAC metastasis might also provide indirect information on the role of *DPC4* in EMT/EMP. A series of studies suggests that functional SMAD4 suppresses metastatic colonization. For instance, an increased number of liver metastases was associated with mutations in *DPC4* rather than a loss of the gene. A SMAD4-negative status correlated with the tendency to metastasize rather than to recur locally, and a genetic subtype of PDAC characterized by biallelic loss of *DPC4* along with a *TP53* missense mutation was linked to higher metastatic efficiency. In a GEMM of PDAC, homozygous loss of *TP73* in *Pdx1-Cre/LSL-Kras^G12D^/Ink4a/Arf^F/F^* mice was associated with a decrease in Smad4 expression, enhanced EMT and migratory activities in PDAC cells isolated from the tumors, and shortened survival of the mice [1]. Here, the increase in EMT and invasion was stimulated by TGF-β and, more specifically, through non-canonical, Smad4-independent signaling [1]. This mechanism, which we have recently confirmed to operate also in human PDAC-derived cells in vitro after RNA interference-mediated knockdown of *TP73* [8], might also explain i) why *DPC4* loss appears to be linked to enhanced metastasis and a worse prognosis and ii) how TGF-β can induce EMT in the absence of SMAD4.

Other studies also indicate that tumors can exhibit EMT when *DPC4* is inactivated. A retrospective clinical study that classified PDAC specimens as being phenotypically epithelial, hybrid or mesenchymal determined *DPC4* loss to be correlated with the mesenchymal phenotype, worse outcome and distant recurrence. Another study observed an association between *DPC4* inactivation and the immune-escape subtype, which was characterized, besides a worse prognosis, by a high rate of tumor budding indicative of EMT [9]. Taken together, the loss of (functional) SMAD4 in PDAC might promote TGF-β-mediated tumorigenesis by abolishing its tumor-suppressive functions while maintaining some tumor-promoting TGF-β responses, i.e., EMT, in part by a switch to non-canonical TGF-β pathways. 

While the existence of EMT in PDAC is well established, that of EMP has recently been evoked in various studies. For instance, PDAC cells can adopt two different EMT programs, a partial (p) or a complete (c) EMT program [10]. The cEMT program is characterized by cells that are derived from poorly differentiated/mesenchymal tumors, transcriptionally repress epithelial markers, such as E-cadherin, enhance mesenchymal markers and migrate in a single-cell mode. In contrast, the pEMT program is characterized by cells that are derived from well-differentiated/epithelial-type PDACs, co-express epithelial and mesenchymal markers, remove E-cadherin from the cell surface by transport to intracellular stores, and migrate in a collective fashion. It has been suggested that cells migrating in clusters, although less motile, have enhanced seeding capacity. This would suggest that tumor cells in pEMT states are more metastatic than cEMT cells, matching the increased aggressiveness of intermediate EMT states mentioned above. Unfortunately, *Dpc4*/*DPC4* expression or mutational status was not evaluated in [10], yet no conclusions on possible differences in its expression in pEMT and cEMT cells or on a possible causative role of SMAD4 in generating these states could be drawn. Interestingly, however, in a study using PDAC organoids, tumor cells utilized different invasion programs depending on *DPC4* status, with collective invasion uniquely present in organoids with *DPC4* loss [11]. Of note, invasion in *DPC4*-mutant organoids required exogenous TGF-β acting through non-canonical signaling via RAC1/CDC42 [11]. These data match the abovementioned observations that PDAC with loss of Smad4 expression due to *TP73* knockout [1] or knockdown [8] are more invasive due to TGF-β-induced activation of non-canonical signaling. To further complicate this scenario, we have recently demonstrated the PDAC line PANC-1, previously classified as quasimesenchymal [12] and as cells with pEMT [10], to consist of various subclones with different EMT phenotypes as indicated by different E-cadherin:vimentin expression ratios and migratory activities [13]. Preliminary analysis indicates that clones possessing a more epithelial-like state express more SMAD4 protein than the more mesenchymal-like EMP states, which is possibly due to higher levels of the RAC1 splice isoform, RAC1b, a positive regulator of SMAD4 expression [8]. Racu et al. speculated that cells presenting with *DPC4* alterations acquire an early pEMT state, closer to an epithelial phenotype and characterized by enhanced metastatic potential. Together, data indicate that *DPC4* inactivation (or SMAD4 protein loss) may play an important role in inducing pEMT states or, in other words, that SMAD4 is a determinant of specific EMP states. 

Altogether, the authors concluded that the biological role of *DPC4* alterations in EMT/EMP of PDAC has not been completely elucidated and that some controversies still exist. They further proposed that the divergent results are explainable by the lack of standardization and criteria to evaluate EMP/EMT. Based on the Consensus Statement mediated by TEMTIA (The EMT International Association), there exist multiple issues concerning the characterization of EMT. To name but a few, (i) EMT cannot solely be defined by the expression of one or few biomarkers but requires diverse markers, or by analysis of only the RNA or protein fraction, since regulation of EMT operates via post-transcriptional regulation at both the mRNA and protein levels, (ii) there are no guidelines as to how to define EMT based on the cellular and molecular markers and heterogeneity exists in the combination of markers used to determine EMT or specific EMP states, (iii) EMT is also characterized by the modulation of cellular properties and characteristics such as the loss of apical-to-basal polarity and increased cell motility, which need to be evaluated in parallel, and (iv) EMT remains a dynamic process with certain EMT phenotypes representing either a definitive or a transient state of the tumor. Finally, it is hypothesized that the disagreements concerning the biological function of SMAD4 in PDAC might be due to the fact that the majority of research on *DPC4* alterations and EMT focused on the binary model of EMT rather than considering the context of EMP.

We agree with the authors that SMAD4 inactivation may play an important role by inducing pEMT or specific EMP states with enhanced metastatic potential, although the biological functions of SMAD4 alterations in EMT/EMP are far from being completely elucidated. Besides the issues regarding faithful characterization of the EMT or EMP processes, this is in part due to the complex role of SMAD4. This protein is located at the crossroads of several signaling pathways that, although similar in architecture, differ by their activating ligands and functional outputs. This is best exemplified within the TGF-β branch, as TGF-β may signal through ALK5 via SMAD2/3/4 and the BMP receptor, ALK2, via SMAD1/5/4 with potential opposing functions on EMT/EMP [14,15]. Hence, it would be interesting to study the different branches of the TGF-β/BMP family and their potential mutual influence on EMT/EMP. We agree with Racu and colleagues in believing that a better understanding of the molecular mechanisms underlying the role of *DPC4* in EMP could help to elucidate its prognostic or diagnostic role, if any, and to facilitate the design of effective targeted therapies in PDAC.
